# Construct Validity of the Late-Life Function and Disability Instrument in African American Breast Cancer Survivors

**DOI:** 10.3390/healthcare4040087

**Published:** 2016-11-16

**Authors:** Ekta Pandya, Jay Mistry, Megha Dobhal, Sujana Borra, Raheem J. Paxton

**Affiliations:** Institute of Healthy Aging, University of North Texas Health Science Center, Fort Worth, TX 76107, USA; ekta.pandya@live.unthsc.edu (E.P.); jam0834@live.unthsc.edu (J.M.); megha.dobhal89@gmail.com (M.D.); sujana.borra@live.unthsc.edu (S.B.)

**Keywords:** African American, breast cancer, cancer survivorship, comorbid conditions, disability, education, functional status, validity, reliability

## Abstract

Limited data exist on the validity of the Late-Life Function and Disability (LLFD) instrument in cancer survivors. We examined the construct validity of the abbreviated LLFD instrument in a sample of African-American breast cancer survivors. African American breast cancer survivors (*n* = 181) aged 50 years and older completed the abbreviated LLFD instrument and questions about sociodemographic and lifestyle characteristics. Confirmatory factor analysis (CFA), Cronbach alphas, and structural models were used to evaluate the construct validity of these measures. Minor modifications were made to the three-factor functional component portion of the inventory to improve model fit. Cronbach alpha’s (range 0.85–0.92) and inter-factor correlations (*r* = 0.3–0.5, all *p* < 0.05) were appropriate. The two-factor disability component fit the data and Cronbach alpha’s (0.91 and 0.98) were appropriate with a high inter-factor correlation (*r* = 0.95, *p* < 0.01). The average variance extracted (range = 0.55–0.93) and composite reliabilities (range = 0.86–0.98) were in acceptable ranges. Floor effects ranged from 7% for advanced lower function to 74% for personal role disability. Education and number of comorbidities were correlated significantly with functional outcomes. The abbreviated LLFD instrument had adequate construct validity in this sample of African American breast cancer survivors. Further studies are needed that examine the stability of the instrument over time.

## 1. Introduction

The recent advances in early detection and treatment of cancer have led to a significant reduction in poor cancer-specific outcomes [1]. Thus, more women are living cancer free with five-year relative survival rates for breast cancer exceeding 95% [2]. However, the survival rates are not distributed uniformly among all cancer survivors, with the rates among African American women trailing behind those of other racial and ethnic groups significantly [2]. While poor screening habits and socio-demographic characteristics predispose minority women to lower survival rates [1], lifestyle characteristics (i.e., obesity, physical inactivity), and deficits in health-related quality of life may contribute equally to disparities in survival [3,4,5,6,7].

Health-related quality of life (HRQOL) consists mainly of mental and physical health domains, which are important determinants of cancer survival. According to the Medical Outcomes Study 36-Item Short-Form Health Survey (SF-36) [8], physical health is operationalized as physical functioning, bodily pain, fatigue (or vitality), and physical role functioning (SF-36). Similarly, mental health is operationalized as social roles, mental health, emotional role functioning, and general health perceptions (SF-36). Of the two domains, physical health, and specifically physical functioning, have been shown to be related to cancer survival [9,10]. Physical functioning includes limitations in mobility, decreases in strength, and limited range of motion in limbs [11,12]. The study of physical functioning is critical not only because it is an important indicator of cancer survival [10], but cancer survivors are also twice as likely to have deficits in functional status when compared to age-matched controls [13]. Of importance, studies have indicated that physical function scores are significantly lower among African American cancer survivors when compared to survivors of other ethnic groups [14]. 

Physical function can be measured using performance-based or self-report measures [15,16]. Short physical performance batteries have been shown to have high reliability and are predictive of disability, institutionalization, and mortality [17,18,19]. However, the objective assessment of physical function is not always feasible and practical as a survey and is more costly [20]. It should also be noted that self-report measures, despite their ease of implementation, are associated with limitations as well. The limitations of self-report instruments are their lack of sensitivity to change, concerns over reproducibility, and inability to capture a broad range of functioning [20]. Moreover, several studies have shown moderate to poor associations between performance-based measures and self-reported functional status. To address the limitations of existing instruments used to assess physical function, the Late-Life Function and Disability (LLFD) Instrument was developed [21,22]. The LLFD instrument is comprised of two components. The functional component assesses advanced lower extremity function (e.g., running 1/2 mile), basic lower extremity function (e.g., getting in and out of a car), and upper extremity function (e.g., holding a full glass of water in one hand) [21]. The disability component assesses the frequency of performing social (e.g., visiting friends or family in their home) and personal (e.g., taking care of household business) role activities and the limitation in the capability of performing instrumental and management role activities [22]. The LLFD instrument is a comprehensive measure of functional status. The advanced lower extremity function subscale represents items requiring reasonable effort, whereas basic lower extremity function represents activities of daily living that are essential to normal functioning [21,22]. Upper body functioning is of relevance to cancer survivors because approximately one-third of the breast cancer survivors suffer from upper extremity disability that could progress to chronic arm disability [23]. More recently, an abbreviated version of the LLFD instrument was proposed as a more parsimonious model and found to have adequate construct, convergent, and predictive validity in an ethnically diverse sample of older adults [24]. 

To date, the LLFD instrument and its abbreviated version have been assessed only in non-cancer samples. We do not know of any studies that have assessed the construct validity of this instrument in adult samples with chronic health conditions. Minority breast cancer survivors experience a greater burden of functional limitations and disability due to poor lifestyle characteristics and late stage of presentation when compared to non-Hispanic white breast cancer survivors. Thus, our sample of underserved African American breast cancer survivors represents a unique sample to examine the validity of this instrument. Therefore, the purpose of this study was to examine the construct validity of the abbreviated LLFD instrument in a sample of African-American breast cancer survivors and examine its association with sociodemographic and lifestyle characteristics.

## 2. Materials and Methods

### 2.1. Recruitment and Participants

African American Breast Cancer Survivors aged 18–70 were identified with the help of the Sisters Network, Inc. (Houston, TX, USA), the largest African-American breast cancer survivorship organization in the United States. The women were recruited via multiple email blasts and the posting of the anonymous survey links on social media and blog sites associated with the Sister Network between May and July of 2012. Messages reached approximately 16,000 members in their database including approximately 3800 breast cancer survivors. Eligibility included: (a) being an African American woman, (b) a previous diagnosis of breast cancer, and (c) being at least 18 years old at time of survey administration. Detailed methods describing our recruitment efforts were reported elsewhere [25]. For the purpose of this study, we focused on women who were at least 50 years old at the time of survey administration (*n* = 181). All subjects consented to participate in the survey, and the study was conducted in accordance with the Declaration of Helsinki. The protocol was approved by the Ethics Committee of the University of North Texas Health Science Center (2013-238).

### 2.2. Measures

#### 2.2.1. Abbreviated Late-Life Function and Disability (LLFD) Instrument

The abbreviated LLFD instrument was utilized to assess discrete functional activities and disability in performing social and personal roles and activities in a physical environment [24]. The functional component was comprised of three subscales assessing advanced lower-extremity function, basic lower-extremity function and upper-extremity function. Each subscale consisted of five items. The 15-items were scored on a Likert-type response scale ranging from 1 (none) to 5 (cannot do). A higher score on the scale represents more difficulty in performing the activity. The disability component was comprised of two subscales assessing personal- and social-role disability. Each subscale consisted of four items which were scored on a Likert-type response scale ranging from 0 (not at all) to 5 (completely). A higher score on the disability component reflects the greater difficulty in performing activities of daily living. 

#### 2.2.2. Socio-Demographic and Medical Data

All socio-demographic and medical data were self-reported by participants. We collected data on the following variables: education (college degree or not), current age, age at cancer diagnosis, disease stage at diagnosis, smoking status, and comorbid conditions. We summed the number of chronic health conditions (e.g., cardiovascular disease, blood sugar/diabetes, digestive disorders, arthritis, and osteoporosis) that were self-reported. Years out from diagnosis was tabulated from current age and age at diagnosis. Self-reported height and weight data were used to calculate participants’ body mass index in a standard manner: weight in kilograms was divided by height in meter squared. 

### 2.3. Statistical Analysis

Initially, descriptive statistics were used to characterize the sample and LLFD instrument constructs. We then investigated the constructs for floor and ceiling effects. Next, a confirmatory factor analysis was used to confirm the construct validity of the instrument. Inter-factor latent variable correlations were used to determine how well the instruments were distinguishable (i.e., discriminate validity) from each other. Furthermore, structural models were computed to examine associations between the instruments, sociodemographic, medical, and lifestyle characteristics.

#### 2.3.1. Confirmatory Factor Analysis (CFA)

Individual CFA models were constructed utilizing full-information maximum likelihood (FIML) estimation to examine the structural validity of the psychosocial constructs using Mplus version 5.21 (Muthen & Muthen, Los Angeles, CA, USA) [26]. Use of the FIML estimator is common in missing data analysis and normally generates better estimates when compared to pairwise or listwise deletion [27]. The extent of missing data ranged from 0% for sociodemographic and medical variables to 7% for disease stage at diagnosis. 

#### 2.3.2. Model Fit

All models are evaluated based on how well structural model resembled close, exact, and absolute fit to the data. According to Hu and Bentler [28], the Comparative Fit Index (CFI) and the Standardized Root Mean Square Residual (SRMR) were optimal for examining structural models with smaller sample sizes. The CFI and SRMR reveal that models are a close fit to the data when values are ≥0.95 and ≤0.08, respectively. Hu and Bentler [28] propose that using cut-off values ≥0.95 for the CFI in combination with values of ≤0.08 for the SRMR results in lower type I and II error rates. Values of average variance extracted (AVE) and composite reliability (CR) were also computed. Recommended ranges for AVE and CR are ≥0.50 and ≥0.70, respectively [29]. 

#### 2.3.3. Model Modification

Modifications to the hypothesized structure were based on substantive and empirical information and face validity as determined by the authors. Modifications were made to the measurement model only when the change resulted in an improved fit, based on a reduction in chi-square value, improved CFI or SRMR values, and if it was theoretically consistent. 

#### 2.3.4. CFA with Covariates

A final model was constructed examining the relationship between study covariates and each of the latent factors. Two separate models were constructs to examine these relationships, which included (a) functional and (b) disability components. Statistical tests were two-sided, and significance was determined at *p* ≤ 0.05.

## 3. Results

### 3.1. Sample Characteristics

Study participants were on average 60 ± 7 years old, 9 ± 7 years out from their most recent cancer diagnosis, and diagnosed with stage I (40%) or II (45%) disease. In addition, most of the women were never-smokers (61%), college educated (55%) and obese (51%). The descriptive statistics of study participants are reported in Table 1. 

### 3.2. Characteristics of Constructs

#### 3.2.1. Function

Summative scores for upper, basic lower, and advanced lower extremity function ranged from five to 25 for all subscales. The mean of upper extremity function was 7.6 ± 3.9, with 38% of the sample scoring the lowest possible value and 1% scoring the highest value. The mean of basic lower extremity function was 7.3 ± 4.2, with 51% scoring the lowest possible value and 2% scoring the highest value. The mean of advanced lower extremity function was 11.8 ± 5.3, with 7% scoring the lowest possible value and 3% scoring the highest possible value. 

#### 3.2.2. Disability

Summative scores for social and personal role disability ranged from 4 to 20 for both subscales. The mean of social role disability was 5.8 ± 3.5, with 65% scoring the lowest possible value and 2% scoring the highest possible value. The mean of personal role disability was 5.8 ± 4.5, with 74% scoring the lowest possible value and 5% scoring the highest possible value. 

### 3.3. Construct Validity

#### 3.3.1. Function

The hypothesized model for the functional component of the LLFD instrument was a marginal fit to the data (*Χ^2^* = 278.1, DF = 87, CFI = 0.92, SRMR = 0.06). Based on the modification indices, we specified a correlation among items three and four of the latent variable pertaining to upper extremity function. This yielded a substantial improvement in the measurement model (*Χ^2^* = 216.5, DF = 86, CFI = 0.94, SRMR = 0.05). The change in chi-square value (Δ*Χ^2^* = 68, relative to the change in DF (ΔDF = 1), exceeded the threshold (i.e., *Χ^2^* (1) = 3.84), indicating that the models were significantly different. The factor loadings for upper body function ranged from 0.56 (i.e., unscrewing the lid off a previously unopened jar without using any devices) to 0.96 (i.e., pouring from a large pitcher). The factor loadings for basic lower function ranged from 0.76 (i.e., using a step stool to reach into a high cabinet) to 0.94 (i.e., picking up a kitchen chair and moving it, in order to clean). The factor loadings for advanced lower function ranged from 0.45 (i.e., running 1/2 mile or more) to 0.85 (i.e., going up and down a flight of stairs outside, without using a handrail). Latent variable correlations among the functional components were statistically significant (All *p* < 0.05). Upper extremity function was modestly correlated with both basic lower (*r* = 0.51, *p* < 0.001) and advanced lower extremity (*r* = 0.31, *p* < 0.001) function, which were both modestly correlated (*r* = 0.40, *p* < 0.001). Internal consistency reliability ranged from 0.85 for advanced lower function to 0.92 for basic lower function. All factor loading were reported in Table 2, whereas latent variable correlations are reported in Table 3.

#### 3.3.2. Disability

The hypothesized model with both personal and social aspects of disability fit the data (*Χ^2^* = 126.2, DF = 19, CFI = 0.95, SRMR = 0.03). No revisions were made to the measurement model. The factor loadings for social role disability ranged from 0.68 (i.e., Invite (Inviting) people into your home for a meal or entertainment) to 0.96 (Go (Going) out with others to public places such as restaurants or movies). The factor loadings for the personal role disability component ranged from 0.95 (i.e., take (taking) care of household business and finances) to 0.98 (i.e., take (taking) care of your own personal care needs). The Chronbach alpha for social and personal role disability was 0.91 and 0.98, respectively, and, the latent variable correlation among the constructs, was highly significant (*r* = 0.95, *p* < 0.01). 

### 3.4. Convergent Validity

Average variance extracted and composite reliabilities were in recommended ranges. The average variance extracted was lowest for advanced lower extremity function (AVE = 0.55) and highest for personal role disability (AVE = 0.93). Composite reliability was lowest for advanced lower extremity function (CR = 0.86) and highest for personal role disability (CR = 0.98). Values of AVE and CR are reported in Table 4.

### 3.5. Association with Covariates

Separate structural models for function and disability were constructed. The structural model examining the sociodemographic, lifestyle, and medical correlates of the disability components fit the data (*Χ^2^* = 162.5, DF = 55, CFI = 0.95, SRMR = 0.03). No covariates were significantly associated with the disability components in the model (All *p* > 0.05). The model accounted for 3% of the variance in both social- and personal-role disability. The structural model examining the sociodemographic, lifestyle, and medical correlates of the functional components fit the data (*Χ^2^* = 314.3, DF = 158, CFI = 0.93, SRMR = 0.05). In the structural model, upper extremity function was significantly associated with education (β = −0.57, *p* < 0.001) and number of comorbid conditions (β = 0.17, *p* = 0.03). Advanced lower extremity function was significantly associated with education (β = −0.55, *p* < 0.001), body mass index (β = 0.32, *p* < 0.001), and number of comorbid conditions (β = 0.22, All *p* = 0.003). Basic lower extremity function was significantly associated with disease stage at diagnosis (β = 0.24, *p* = 0.024), education (β = −0.54, *p* < 0.001), and number of comorbid conditions (β = 0.20, *p* = 0.009). No other associations were observed (Table 5). The structural model accounted for 13% of the variance in upper extremity function, 16% of the variance in basic lower extremity function, and 28% of the variance in advanced lower extremity function. 

## 4. Discussion

The primary purpose of this study was to examine the construct validity of the abbreviated LLFD instrument in a sample of African-American breast cancer survivors. We found that the LLFD instrument fit the data with minor modifications. These data support prior studies examining the LLFD instrument in an older adult population and potentially extends the validity of the instrument to cancer survivors. In addition, we observed that education and number of comorbid conditions were associated significantly with all measures of function. Overall, these data demonstrate adequate construct validity of the LLFD instrument in a vulnerable population of cancer survivors but also sheds some light on sub-populations within African American breast cancer survivors who deserve special attention for interventions to remedy functional limitations. 

Minor modifications were made to improve the fit of the functional component of the LLFD instrument. In particular, we added a residual variance correlation among two items representing upper extremity functioning (i.e., Item #3: using common utensils for preparing meals; Item #4 holding a full glass of water in one hand). These items have similar conceptual meaning as both of them relate to working with one’s hands. Despite the minor modifications, the functional subscales demonstrated simple structure with high factor loadings indicative of strong construct validity. Thus, we feel that our results support those observed in prior studies using this abbreviated instrument [24]. The brevity of functional components and its demonstrated reliability and validity is of importance given prior studies indicating that functional status was associated with cancer-specific and overall survival [10,30]. Importantly, this instrument has relevance for cancer survivors due to the variability in the scores and low frequency of floor and ceiling effects. Prior studies have demonstrated that the LLFD instrument was sensitive to change and may serve as a self-report outcome of functional status in intervention studies [20].

Our results also confirm the construct validity of the social and personal components of the disability instrument. The instrument had adequate factor loadings and was correlated significantly with the functional components, similar to a prior study [24]. In addition, there a sizable portion (>50%) of the sample who reported scores at the lowest end indicating a large floor effect. This may also suggest that most of the survivors participating here had reasonable levels of function and therefore few disabilities. Of note, the sociodemographic, medical, and lifestyle characteristics were not associated with the disability subscales. This could be due to the lack of variance in the responses as a sizable portion of women reporting the lowest possible scores on the subscales. More research is needed to determine how well these items correlate with objective measures of function and disability in survivor populations. 

Further evidence to support the construct validity of the abbreviated LLFD instrument were the associations we observed between the sociodemographic, medical, and lifestyle characteristics with the functional components. In particular, less education was associated with worse upper and lower extremity function. The associations we observed among our survivors are similar to those found in prior studies because education is a proxy for socioeconomic status and thus indicates earning power or lack thereof [31,32,33]. Becker et al. [34] observed that comorbidities were associated with not only physical and functional health but also social and emotional health as well. The associations we observed here may point to high-risk groups that can be targeted for interventions. African American breast cancer survivors who are less educated may be more likely to have co-occurring conditions such as high blood pressure and diabetes and may be more vulnerable to functional limitations due to their disease state. 

The results from this study provide noteworthy and unique information about a vulnerable population of cancer survivors. Strengths of our study include a modest sample size of underrepresented cancer survivors as well as the use of an instrument not commonly examined in the field of cancer survivorship. Despite the strengths, several limitations should be noted. Our data are cross-sectional and therefore can not imply causality. Our sample size is modest and consists of mostly affluent African American breast cancer survivors, limiting the generalizability of our study. In addition, our data on functional limitations are self-reported and subject to reporting biases. Further studies will be needed to compare the abbreviated LLFD instrument in cancer survivors to objective measures of functional status. 

## 5. Conclusions

Overall, this is the first study to our knowledge to examine the construct validity of the abbreviated LLFD instrument in a sample of cancer survivors. Importantly, our sample is of interest because they experience greater levels of functional deficits and disability when compared to survivors of other ethnic groups. Our data show that the abbreviated LLFD instrument has adequate validity in African American breast cancer survivors. Studies are needed to examine the stability of the instrument over time and the predictive validity of the instrument to objective measures of functional status in the African American breast cancer survivors. Furthermore, more research is needed to identify vulnerable minority cancer survivors who will benefit from lifestyle interventions designed to improve functional status. Such studies may not only improve functional status, but reduce survival disparities that exist between non-Hispanic White and African American cancer survivors. 

## Figures and Tables

**Table 1:** Demographic characteristics of sample (*n* = 181).

Variable	SD or %
Mean Age (SD)	59.7 (7.2)
Mean age at diagnosis (SD)	51.0 (8.0)
Disease stage at diagnosis, *n* (%)	
I	68 (40%)
II	75 (45%)
III	25 (15%)
Missing	13
Education, *n* (%)	
% College Graduate	99 (55%)
Lifestyle Variables	
Mean body mass index (SD)	30.8 (5.8)
Body size, *n* (%)	
Normal weight	30 (16%)
Overweight	59 (33%)
Obese	92 (51%)
Smoking Status	
Never smoker	110 (61%)
Mean number of comorbidities (SD)	1.6 (1.1)

SD = Standard Deviation.

**Table 2:** Factor loading and internal consistency reliability coefficients for the Late-Life Function and Disability (LLFD) Components.

Function	Upper	Basic Lower	Advanced Lower
(1) Unscrewing the lid off a previously unopened jar without using any devices	0.59	-	-
(2) Running 1/2 mile or more	-	-	0.45
(3) Using common utensils for preparing meals (e.g., can opener, potato peeler)	0.82	-	-
(4) Holding a full glass of water in one hand	0.73		-
(5) Walking a mile, taking rests as necessary	-	-	0.77
(6) Going up and down a flight of stairs outside, without using a handrail	-	-	0.85
(7) Ripping open a package of snack food using only your hands	0.81	-	-
(8) Pouring from a large pitcher	0.96	-	-
(9) Getting into and out of a car/taxi	-	0.85	-
(10) Going up and down three flights of stairs inside, using a handrail	-		0.79
(11) Picking up a kitchen chair and moving it, in order to clean	-	0.94	-
(12) Using a step stool to reach into a high cabinet	-	0.76	-
(13) Carrying something in both arms while climbing a flight of stairs (e.g., laundry basket)	-	-	0.78
(14) Bending over from a standing position to pick up a piece of clothing from the floor	-	0.82	-
(15) Walking around one floor of your home, taking into consideration doors, furniture, and a variety of floor coverings	-	0.92	-
Internal Consistency Reliability	0.89	0.92	0.85
**Disability**	**Social Role**	**Personal Role**	-
(1) Visit (Visiting) friends and family in their homes	0.89		-
(2) Take (Taking) care of household business and finances	-	0.95	-
(3) Travel (Traveling) out of town for at least an overnight stay	0.81	-	-
(4) Invite (Inviting) people into your home for a meal or entertainment	0.68	-	-
(5) Go (Going) out with others to public places such as restaurants or movies	0.96	-	-
(6) Take (Taking) care of your own personal care needs	-	0.98	-
(7) Take (Taking) care of local errands	-	0.96	-
(8) Prepare (Preparing) meals for yourself	-	0.96	-
Internal Consistency Reliability	0.91	0.98	-

**Table 3:** Latent variable correlation coefficients among the Late-Life Function and Disability components.

	Upper Body	Advanced Lower	Basic Lower	Social Role	Personal Role
Upper Body	1.0				
Advanced Lower	0.48 **	1.0			
Basic Lower	0.64 **	0.63 **	1.0		
Social Role	0.25 **	0.23 **	0.25 **	1.0	
Personal Role	0.39 **	0.21 **	0.33 **	0.80 **	1.0

** *p* < 0.01. Spearman correlation coefficients were derived from summative scores of the function and disability components.

**Table 4:** Convergent validity statistics for the Late-Life Function and Disability instrument.

	Average Variance Extracted	Composite Reliability
Upper Body	0.64	0.90
Advanced Lower	0.55	0.86
Basic Lower	0.74	0.93
Social Role	0.71	0.91
Personal Role	0.93	0.96

**Table 5:** The association between Late-Life Functional Disability Components and study covariates.

	Upper Body	Advanced Lower	Basic Lower	Social Role	Personal Role
Age	0.07	0.10	0.12	−0.00	−0.02
Disease stage at diagnosis	0.20	0.13	0.24 *	0.12	0.17
Years out from diagnosis	−0.02	−0.01	−0.15	−0.07	−0.02
Education	−0.57 **	−0.55 **	−0.54 **	−0.21	−0.17
Body mass index	0.12	0.32 **	0.05	0.04	0.10
Number of comorbid conditions	0.17 *	0.22 **	0.20 **	0.03	−0.01

* *p* < 0.05; ** *p* < 0.01; Coefficients represents standardized Beta coefficients observed in models with latent factors regressed on covariates.

## Abbreviations

The following abbreviations are used in this manuscript:

LLFD	Late-Life Function and Disability
CFA	Confirmatory factor analysis
HRQOL	Health-related quality of life
FIML	Full information maximum likelihood
CFI	Comparative fit index
SRMR	Standardized root mean square residual
DF	Degrees of freedom
AVE	Average variance extracted
CR	Composite reliability
